# Severe metabolic acidosis after out-of-hospital cardiac arrest: risk factors and association with outcome

**DOI:** 10.1186/s13613-018-0409-3

**Published:** 2018-05-08

**Authors:** Matthieu Jamme, Omar Ben Hadj Salem, Lucie Guillemet, Pierre Dupland, Wulfran Bougouin, Julien Charpentier, Jean-Paul Mira, Frédéric Pène, Florence Dumas, Alain Cariou, Guillaume Geri

**Affiliations:** 1 Medical Intensive Care Unit, Cochin University Hospital (APHP), 27 rue du Faubourg Saint Jacques, 75014 Paris, France; 2 Emergency Department, Cochin University Hospital (APHP), Paris, France; 3 Paris Descartes University, Paris, France; 4 INSERM U970, Sudden Death Expertise Centre, Paris Cardiovascular Research Centre, Paris, France

**Keywords:** Metabolic acidosis, Out-of-hospital cardiac arrest, Post-resuscitation syndrome, Outcome

## Abstract

**Background:**

Metabolic acidosis is frequently observed as a consequence of global ischemia–reperfusion after out-of-hospital cardiac arrest (OHCA). We aimed to identify risk factors and assess the impact of metabolic acidosis on outcome after OHCA.

**Methods:**

We included all consecutive OHCA patients admitted between 2007 and 2012. Using admission data, metabolic acidosis was defined by a positive base deficit and was categorized by quartiles. Main outcome was survival at ICU discharge. Factors associated with acidosis severity and with main outcome were evaluated by linear and logistic regressions, respectively.

**Results:**

A total of 826 patients (68.3% male, median age 61 years) were included in the analysis. Median base deficit was 8.8 [5.3, 13.2] mEq/l. Male gender (*p* = 0.002), resuscitation duration (*p* < 0.001), initial shockable rhythm (*p* < 0.001) and post-resuscitation shock (*p* < 0.001) were associated with an increased level of acidosis. ICU mortality rate increased across base deficit quartiles (39.1, 59.2, 76.3 and 88.3%, *p* for trend < 0.001), and base deficit was independently associated with ICU mortality (*p* < 0.001). The proportion of CPC 1 patients among ICU survivors was similar across base deficit quartiles (72.8, 67.1, 70.5 and 62.5%, *p* = 0.21), and 7.3% of patients with a base deficit higher than 13.2 mEq/l survived to ICU discharge with complete neurological recovery.

**Conclusion:**

Severe metabolic acidosis is frequent in OHCA patients and is associated with poorer outcome, in particular due to refractory shock. However, we observed that about 7% of patients with a very severe metabolic acidosis survived to ICU discharge with complete neurological recovery.

**Electronic supplementary material:**

The online version of this article (10.1186/s13613-018-0409-3) contains supplementary material, which is available to authorized users.

## Background

Among successfully resuscitated out-of-hospital cardiac arrest (OHCA) patients admitted to the intensive care unit (ICU) after return of spontaneous circulation (ROSC), the occurrence of a “post-cardiac arrest syndrome” is the leading cause of early mortality. This syndrome is related to multi-organ ischemic reperfusion injury and may lead to multi-organ failure in the early hours after resuscitation [1].

Acidosis is frequently observed in this context [2] and is often multifactorial, resulting from major metabolic disturbances (increased levels of blood lactate, phosphate, unmeasured anions, acute kidney injury) as well as respiratory disturbances. Metabolic acidosis has well-known deleterious effects including decrease of cardiac contractility [3], arterial vasodilatation [4], impairment of the inflammatory and immune response [5] leading to an increased risk of multi-organ failure. Accordingly, a strong association between the depth of acidosis and poor outcome has been previously demonstrated in critically ill patients [6, 7]. Even though intuitive in OHCA patients, data on the prevalence and the severity of metabolic acidosis at ICU admission are scarce. Moreover, data on the prognostic impact of metabolic acidosis in these patients and on the proportion of patients with severe metabolic acidosis discharged alive from ICU are lacking.

In the present study, we aimed to describe the outcome of successfully resuscitated OHCA patients with metabolic acidosis and to identify factors associated with severe acidosis and survival to ICU discharge.

## Methods

### Study population

In the present analysis, we screened all consecutive OHCA patients admitted to our cardiac arrest center between January 2007 and December 2012 (IRB number CE-SRLF 12-384). We then selected patients who evidenced a metabolic acidosis at ICU admission, defined by a positive base deficit, as described above. We only excluded patients who did not get ROSC in the prehospital setting and who received CPR at time of ICU admission.

### Data collection and definitions

Cardiac arrest characteristics, in-hospital management and outcome data were prospectively collected according to the Utstein style [8]. The following information was recorded prospectively for each patient: demographic data, clinical parameters, cardiac arrest location, time from collapse to basic life support (BLS) and time from BLS to ROSC, initial rhythm, admission blood lactate level, temperature management and ICU mortality. Post-resuscitation shock was defined as the need for vasopressors (epinephrine or norepinephrine) lasting more than 6 h despite adequate fluid loading or the need for mechanical circulatory assistance (intra-aortic balloon pump) [9]. We reviewed patients’ charts in order to collect information that could impact ICU admission bicarbonate level, i.e., the use of intravenous bicarbonate during resuscitation, the existence of chronic respiratory condition and chronic kidney disease in the past medical history.

Laboratory values were computed from medical files and extracted from the patient data management system (Clinisoft, GE Healthcare). We specifically collected arterial blood gas (pH, PCO_2_ [mmHg], PO_2_ [mmHg], bicarbonate [mmol^−1^]), arterial blood lactate, hemoglobin, potassium, phosphorus, blood creatinine and urea levels. We determined the occurrence and the severity of acute kidney injury within the first 48 h using the KDIGO definition [10].

Metabolic acidosis was defined by a positive base deficit (i.e., negative base excess). Base deficit was calculated according to the Van Slyke equation [11, 12] and expressed in mEq/l. We then divided the study population in quartiles of base deficit.

### Early management

As previously described [13], our local practices include a strategy of early imaging work-up performed within the first 24 h after an immediate assessment of the feasibility of further investigations by the Emergency Medical Services (EMS) and ICU physicians. According to this strategy, we consider immediate coronary angiography in all patients without obvious extra-cardiac cause of cardiac arrest, regardless of the initial rhythm and ECG changes. In case of suspected extra-cardiac cause and in the absence of an obvious etiology, a CT scan can also be performed at admission (brain CT scan and chest CT pulmonary angiography). After this early imaging procedure, patients are then admitted to ICU. Arterial blood gas is taken as soon as possible, i.e., either at the cath laboratory arrival, or at ICU admission if the patient was directly admitted to the ICU. Renal replacement therapy is initiated at ICU admission in case of severe metabolic acidosis (defined by a pH lower than 7.20 and an admission bicarbonate level lower than 20 mmol/l) and/or in case of life-threatening hyperkalemia (defined by blood potassium level higher than 6 mmol/l with electrocardiographic findings suggestive of hyperkalemia) [2]. Therapeutic hypothermia was performed using surface cooling, using a 33 °C temperature target.

### Outcomes

The main outcome was survival at ICU discharge. Neurological outcome and cause of death were our secondary outcomes. The cause of death was defined for each non-survivor as related to post-cardiac arrest shock, when death occurred as a direct consequence of shock (including subsequent multi-organ failure), or related to neurological injury if this led to withdrawal of life-sustaining therapy or brain death [9]. Neurological outcome at ICU discharge was assessed using the Cerebral Performance Categories score [14]. Briefly, CPC score ranges from 1 to 5: 1 is a normal neurological state while 4 means vegetative state and 5 death.

### Statistical analysis

Continuous variables were presented as median [interquartile] and categorical variables as counts (percentages). Baseline characteristics were compared according to the presence of acidemia using Mann–Whitney test, and Pearson Chi-square test or the Fisher’s exact test, as appropriate, for continuous and categorical variables, respectively. Characteristics between quartiles of base excess were compared using Cuzick test and trend Pearson Chi-square test for continuous and categorical variables, respectively.

To evaluate prognostic performance of base deficit, admission pH, arterial lactate and bicarbonate level, we picked up thresholds corresponding to a specificity of 100% (i.e., no survivor above the threshold).

Factors associated with base excess were identified using a multivariable linear regression. Normal distribution of residuals was checked as well as heteroscedasticity. Factors associated with ICU mortality were assessed using a multivariable logistic regression. The multivariable model was built using all variables significantly associated with ICU mortality in univariate analysis (*p* < 0.05). The goodness-of-fit of the model was evaluated using the Hosmer–Lemeshow test.

This analysis was repeated in the following subgroups of patients, as sensitivity analyses: patients without prehospital bicarbonate infusion and patients without either chronic respiratory disease or chronic kidney disease. We also performed a sensitivity analysis including arterial blood lactate level in the multivariable model to assess a potential interaction between lactate level and base deficit. We thus included a cross-produced factor, and both lactate level and base deficit were included as continuous variables in the model.

All statistical tests were two-sided using a type I error of 0.05 unless otherwise mentioned. Analyses were performed using Stata 14.1 (Stata Corp, College Station, TX).

## Results

A total of 899 resuscitated OHCA patients were admitted to the study hospital during the study period. We excluded 27 patients without ROSC at ICU admission, one patient with DNR order and 2 patients with no available arterial blood gases at ICU admission. Among the 869 remaining patients with acid–base status available, 43 patients did not have metabolic acidosis, and we included 826 patients in the analysis.

### Baseline characteristics of the studied population

Most of the included patients were male (68.3%) of median age of 61 [iqr 50–73] years (Table 1). An initial shockable rhythm was observed in 414 (50.1%) cases, and the median time from collapse to ROSC was 20 [iqr 13–30] min. Post-resuscitation shock occurred in 480 (58.1%) cases. Acute kidney injury (AKI) was observed in 548/791 (69.3%) patients (baseline creatinine level was unknown in 35/45 patients with chronic kidney disease). Renal replacement therapy (RRT) was initiated within the first 24 h in 40.6% of cases. Even if indicated, 13 patients did not receive RRT because of major hemodynamic instability (*n* = 10) or early withdrawal of life-sustaining therapy (*n* = 3).

**Table 1:** Baseline characteristics of the 826 patients included in the study according to quartiles of base deficit

Variable	All patients *n* = 826	Base deficit quartiles				*p* value
		[0–5] *n* = 207	[5–9] *n* = 206	[9–13] *n* = 207	> 13 *n* = 206	
*Demographics*						
Male gender	564 (68.3)	156 (75.4)	148 (71.8)	139 (67.1)	121 (58.7)	< 0.001
Age (year)	61 [50, 73]	60 [51, 71]	64 [51, 77]	60 [49, 72]	62 [51, 74]	0.678
*OHCA characteristics*						
Public setting	262 (31.8)	79 (38.3)	71 (34.5)	70 (33.8)	42 (20.4)	< 0.001
Witnessed CA	698 (87.0)	181 (90.5)	183 (92.0)	177 (88.5)	157 (77.3)	< 0.001
Bystander CPR	444 (55.8)	122 (62.2)	122 (60.7)	99 (49.5)	101 (51.0)	0.004
Initial VF/VT	414 (50.1)	129 (62.3)	121 (58.7)	92 (44.4)	72 (35.0)	< 0.001
Collapse to ROSC, min	20 [13, 30]	16 [10, 25]	20 [14, 27]	22 [15, 33]	29 [20, 40]	< 0.001
Prehosp. infusion of bicar.	113 (13.7)	17 (8.2)	22 (10.7)	28 (13.5)	46 (22.3)	< 0.001
Chronic respiratory disease	42 (5.1)	11 (5.3)	14 (6.8)	8 (3.9)	9 (4.4)	0.399
Chronic kidney disease	45 (5.5)	5 (2.4)	12 (5.9)	14 (6.9)	14 (7.0)	0.039
*Biological characteristics at ICU admission*						
pH	7.22 [7.11, 7.31]	7.33 [7.28, 7.39]	7.27 [7.21, 7.32]	7.18 [7.13, 7.25]	7.03 [6.92, 7.11]	< 0.001
PCO_2_ (mmHg)	42.8 [36.0, 51.2]	41.5 [36.8, 49.7]	42.3 [36.3, 50.5]	43.5 [35.7, 52.3]	43.5 [35.1, 52.5]	0.554
Bicarbonate level (mmol/l)	17.5 [14.0, 20.4]	21.8 [20.7, 22.9]	19.1 [17.8, 20.0]	16.0 [15.0, 16.9]	10.8 [8.7, 12.7]	< 0.001
Urea level (mmol/l)	7.3 [5.7, 10.2]	6.8 [5.3, 8.9]	7.3 [5.8, 9.8]	7.5 [5.8, 10.6]	8.1 [5.7, 13.2]	< 0.001
Creatinine level (µmol/l)	106 [78, 146]	87 [69, 111]	102 [77, 130]	114 [86, 148]	136 [101, 190]	< 0.001
Phosphorus level (mmol/l)	1.7 [1.1, 2.5]	1.2 [0.9, 1.7]	1.4 [1.0, 1.9]	1.9 [1.3, 2.5]	2.9 [2.1, 3.8]	< 0.001
Lactate level (mmol/l)	5.2 [2.5, 9.2]	2.5 [1.6, 4.3]	4.0 [2.3, 5.7]	6.5 [3.5, 8.8]	11.2 [7.6, 15.0]	< 0.001
Base deficit (mEq/l)	8.8 [5.3, 13.2]	3.6 [2.8, 4.5]	6.8 [6.0, 7.9]	10.8 [9.7, 11.9]	17.8 [15.2, 21.0]	< 0.001
*In*-*hospital characteristics*						
Cardiac cause-related CA	436 (56.2)	136 (68.7)	122 (61.3)	103 (54.5)	75 (39.5)	< 0.001
Post-resus. shock	480 (58.1)	87 (42.0)	109 (52.9)	119 (57.5)	165 (80.1)	< 0.001
Acute kidney injury^a^						< 0.001
No AKI	243 (30.7)	104 (51.5)	71 (35.9)	59 (30.3)	9 (4.6)	
KDIGO 1	65 (8.2)	12 (5.9)	27 (13.6)	19 (9.7)	7 (3.6)	
KDIGO 2	45 (5.7)	17 (8.4)	13 (6.6)	10 (5.1)	5 (2.6)	
KDIGO 3	438 (55.4)	69 (34.2)	87 (43.9)	107 (54.9)	175 (89.7)	
Coronary angiography	556 (67.3)	145 (70.0)	150 (72.8)	135 (65.2)	126 (61.2)	0.019
Therapeutic hypothermia	717 (86.8)	186 (89.9)	182 (88.3)	185 (89.4)	164 (79.6)	0.005
RRT at day-1	335 (40.6)	42 (20.4)	56 (27.2)	89 (43.0)	148 (71.8)	< 0.001

*OHCA* out-of-hospital cardiac arrest, *CPR* cardiopulmonary resuscitation, *ROSC* restoration of spontaneous circulation, *AKI* acute kidney injury, *KDIGO* kidney disease improving global outcome, *RRT* renal replacement therapy

*p* value for trend has been calculated using a Chi-square trend test for binary variables and Cuzick test for ordinal and continuous variables

^a^Missing data are related to the missingness of basal level of creatinine in patients with chronic kidney disease

### Factors associated with severity of metabolic acidosis

Arterial acidemia (pH < 7.38) was observed in the majority of patients at ICU admission (n = 743, 90.0%) with a median pH at ICU admission at 7.22 [7.11, 7.31] and a blood bicarbonate level of 17.5 [14.0, 20.4] mmol/l. Median base deficit was 8.8 [iqr 5.3, 13.2] mEq/l (Additional file 1: Figure S1). Median base deficit was 3.3 [iqr 2.5, 5.3] and 9.5 [iqr 5.9, 13.8] in patients without and with acidemia (*p* < 0.001). There were, respectively, 61, 19, 2 and 0 patients without acidemia in base deficit quartiles. The proportion of witnessed cases and of initial shockable rhythm decreased across base deficit quartiles while time interval from collapse to ROSC increased across the quartiles (16, 20, 22 and 29 min, *p* for trend < 0.001) (Table 1). Left ventricular ejection fraction did not differ in the 4 quartiles of base deficit (median = 40% in the four quartiles). In multivariable linear regression, male gender (beta estimate − 1.51 [95% confidence interval − 2.45, − 0.56]) and initial shockable rhythm (− 2.36 [− 3.41, − 1.31]) were negatively associated with base deficit. Conversely, time interval between collapse to ROSC (+ 0.09 per min [0.06, 0.12]) and the occurrence of post-resuscitation shock (+ 2.45 [1.60, 3.29]) was independently associated with base deficit (Table 2).

**Table 2:** Factors associated with base deficit in multivariable linear regression

Variable	Coefficient	95% confidence interval	*p* value
Age, per year	− 0.01	− 0.03, 0.02	0.670
Male gender	− 1.51	− 2.45, − 0.56	0.002
Public setting	− 0.85	− 1.76, 0.06	0.067
Witnessed CA	− 1.35	− 2.91, 0.22	0.091
Bystander CPR	0.69	− 0.17, 1.56	0.117
Collapse to ROSC, per min	0.09	0.06, 0.12	< 0.001
Initial VF/VT	− 2.36	− 3.41, − 1.31	< 0.001
Post-resus. shock	2.45	1.60, 3.29	< 0.001
Cardiac cause-related CA	− 0.16	− 1.21, 0.89	0.761

*CA* cardiac arrest, *CPR* cardiopulmonary resuscitation, *ROSC* restoration of spontaneous circulation

### Association between base deficit, ICU mortality and cause of death

Overall, 543 (65.7%) patients died in ICU. ICU mortality rate increased across base deficit quartiles (39.1, 59.2, 76.3 and 88.3%, *p* for trend < 0.001). ICU mortality was 42.2 versus 68.4% in patients without and with acidemia (*p* < 0.001). After adjustment for confounders, we observed an association between base deficit and mortality at ICU discharge (odds ratio 1.76 [1.07, 2.91], 3.82 [2.20, 6.65], 5.13 [2.67, 9.88] for 5–9, 9–13 and > 13 categories with base deficit < 5 as reference category, respectively) (Table 3). Similar results were obtained in patients without prehospital bicarbonate infusion and without either chronic respiratory disease or chronic kidney disease. We observed a strong interaction between base deficit and lactate level (*p* for interaction = 0.004).

**Table 3:** Factors associated with ICU mortality in multivariable logistic regression

Variable	Odds ratio	95% confidence interval	*p* value
Age (year)	1.03	1.02, 1.05	< 0.001
Male gender	1.39	0.87, 2.22	0.164
Public setting	0.63	0.41, 0.97	0.034
Witnessed CA	0.96	0.39, 2.35	0.931
Bystander CPR	0.76	0.50, 1.16	0.200
Initial VF/VT	0.43	0.26, 0.71	0.001
Collapse to ROSC, per min	1.05	1.03, 1.07	< 0.001
Cardiac cause-related CA	0.48	0.29, 0.80	0.005
Post-resus. shock	1.31	0.89, 1.96	0.181
Base deficit quartiles (mEq/l)			
< 5	1.00	1.00, 1.00	
5–9	1.76	1.07, 2.91	0.026
9–13	3.82	2.20, 6.65	< 0.001
> 13	5.13	2.67, 9.88	< 0.001

CA cardiac arrest; CPR cardiopulmonary resuscitation; ROSC restoration of spontaneous circulation

**Fig. 1:**
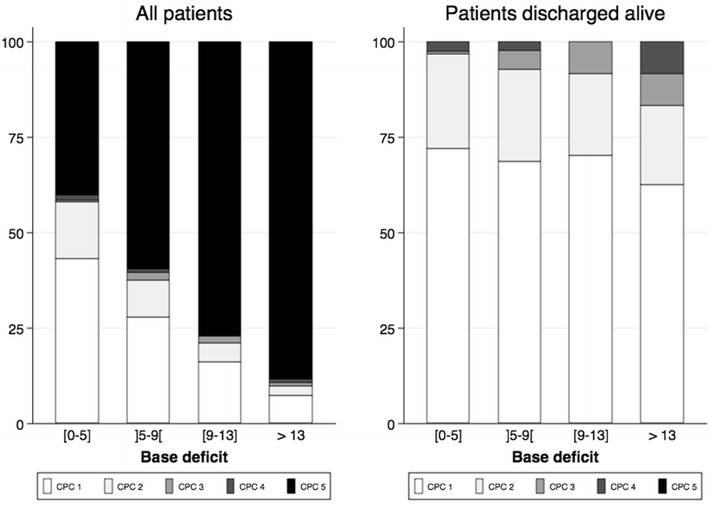
Neurological outcome at ICU discharge in all patients (left panel) and in patients discharged alive from ICU (right panel). CPC, cerebral performance category scale

Brain damage was the leading cause of death (*n* = 367, 67.6%), while refractory shock was responsible for 173 (31.9%) deaths. Three patients died in ICU from septic shock without brain anoxic injury. Death related to refractory shock was more frequent in the subgroup of patients with the most severe metabolic acidosis (13.6, 22.1, 28.5 and 49.5% across base deficit quartiles). Overall, the proportion of CPC1 patients decreased across the base deficit quartile while it was similar among patients discharged alive (72.8, 67.1, 70.5 and 62.5%, *p* = 0.21) (Fig. 1). Among patients with the most severe acidosis, clinical characteristics except initial rhythm and in-hospital management were similar between patients who died and those who were discharged alive from ICU (Additional file 2: Table S1).

No patient survived with a base deficit higher than 25 mmol/l, a pH lower than 6.72, a blood bicarbonate level lower than 8.6 mmol/l or an admission arterial blood lactate level higher than 20 mmol/l (Additional file 3: Table S2).

## Discussion

In the present study, we found that metabolic acidosis was almost constantly observed at ICU admission in OHCA patients. Metabolic acidosis was related to the initial rhythm and the time interval from collapse to ROSC. Furthermore, we observed that metabolic acidosis was associated with ICU mortality in a severity-dependent manner and was also associated with refractory shock. However, we also found that about 10% of patients with a very severe metabolic acidosis (median base deficit > 13.2 mEq/l) survived to ICU discharge with good neurological recovery.

In the present study, metabolic acidosis was severe and strongly determined by the initial cardiac rhythm and the time interval from collapse to ROSC. While several studies have already reported the occurrence of acidosis after cardiac arrest, the present study reports findings from a large cohort of OHCA patients using a reproducible and reliable definition of metabolic acidosis [15–18]. To the best of our knowledge, the present study is the largest study focusing on the prevalence and the prognostic impact of metabolic acidosis in successfully resuscitated OHCA patients. Moreover, several studies reported metabolic acidosis using either pH and/or blood lactate level [19, 20]. This probably includes some patients with exclusive respiratory acidosis, especially in countries where there is no use of advanced airway in the field, and simultaneously misses some patients with compensated metabolic acidosis. This is the reason why we used the base deficit calculation without using pH. Furthermore, the association we observed between severity of metabolic acidosis and both resuscitation duration and initial rhythm may reflect the goodness-of-fit of such a measure with the degree and the duration of inadequate perfusion [21]. Thus, we showed that lactate level was an important contributor of metabolic acidosis but not sufficient to exhaustively describe it [22, 23]. In the present study, acidosis strongly correlated with both lactate level and phosphorus level, highlighting the fact that metabolic acidosis after OHCA is not only related to increase of lactate level but also to accumulation of unmeasured anions partially because of frequent acute kidney injury. This additional finding may explain the association we observed between severity of metabolic acidosis and ICU mortality as acute kidney injury has been demonstrated as an independent marker of ICU mortality after out-of-hospital cardiac arrest [2, 24].

Our findings support a strong association between severity of metabolic acidosis at ICU admission after OHCA and ICU mortality. Interestingly, we were able to provide cause of death and observed that ICU mortality in the most severe patients included in the present study was mostly related to refractory multi-organ failure. The association between metabolic acidosis and ICU mortality has been reported in different subgroups of critically ill patients: trauma [25], sepsis [26] as well as cardiac arrest [27–29]. Nolan et al. [27] reported a significant increase of 45% of in-hospital mortality per 0.1 point of pH decrease below 7.25. In the same manner, Chien et al. [29] reported an odds ratio of survival to hospital discharge of 10 for patients with a pH higher than 7.07 [95% confidence interval 2.1, 47.7]. Although pH is a simple measure to obtain at ICU admission, it does not distinguish metabolic and respiratory acidosis. Indeed, PCO_2_ increase after ROSC may largely contribute to acidemia [30, 31]. Thus, we chose to consider base deficit, more likely to reflect metabolic acidosis and ischemia. We evidenced a severity-manner relationship between base deficit and survival to hospital discharge. Physicians may be aware that pH may add information to base deficit, as we observed that patients with acidemia had worse prognosis than those without. These findings are fully consistent with those previously published in some smaller cohorts [17, 28, 32, 33]. Besides the correlation between base deficit and duration of resuscitation efforts, metabolic acidosis may be associated with mortality by its hemodynamic effects, as depression of ventricular function, catecholamine release and reduction of ventricular responsiveness to catecholamines [4]. Interestingly, we did not evidence any relationship between metabolic acidosis and myocardial function, assessed by the left ventricle ejection function. This might suggest that in our cohort, metabolic acidosis may worsen vasoplegia in a more intensive manner and explain the increase of deaths related to refractory shock we observed across the base deficit quartiles.

We observed a survival rate of about 10% in the fourth quartile of base deficit, i.e., in patients with the most severe metabolic acidosis. In 1985, Weil et al. did not observe any survivor with a pH below 7.25 and an arterial lactate level higher than 7 mmol/l. However, this study was published 30 years ago and in-hospital management of successfully resuscitated OHCA patients has greatly improved since this publication [34]. In the study reported by Chien et al., the lowest pH associated with survival was 6.86 while Nolan et al. reported a survival rate of less than 1% below 6.70, about 3% between 6.70 and 6.80 and about 7% between 6.80 and 6.90 [27, 29]. Recently, Ilicki et al. [35] described 6 OHCA survivors with an initial pH lower than 6.90. However, among these cases, 3 had another underlying cause of acidemia (either ketoacidosis or metformin intoxication), and 2 had hemorrhagic shock. The comparison according to vital status at ICU discharge in this subgroup of patients did not evidence any in-hospital factors that could be associated with survival. We were particularly interested in the similar rate of renal replacement therapy in these patients regardless of the vital status at ICU discharge. Despite acidosis severity (almost three quarters of patients received renal replacement therapy within the first 24 h), several patients survived without aggressive treatment of metabolic acidosis and we did not find any additional factor that could differentiate these patients. Moreover, prehospital infusion of bicarbonate appeared to be strongly correlated with ICU mortality and seemed to be a surrogate marker of the severity assessed by the prehospital team.

We acknowledge several limitations in the present study. First, due to the retrospective design, we are not able to confirm the causality of the association we observed. Despite adjustment for prehospital and in-hospital confounders, we cannot be sure that metabolic acidosis might only be a mediator rather than an independent predictor of ICU mortality. Second, we specifically collected information about acidosis of OHCA patients included in analysis previously performed that focused on the prognostic impact of acute kidney injury in this setting [3]. Thus, we were not able to extend the study period beyond 2012. However, we do believe the results we observed would be similar in a more recent cohort. Third, we were not able to collect additional information on the pathogenesis of the metabolic acidosis. We did not collect chloremia, albumin or magnesium level, which could have allowed us to explore more deeply metabolic acidosis mechanisms. Fourth, we did not collect repeated measurements of base deficit, which prevented us to evaluate the prognostic impact of metabolic acidosis correction or acidosis duration. Last, we did not collect renal replacement therapy details, which could partly explain the differences between survivors and patients who died in the fourth quartile of base deficit.

## Conclusion

Metabolic acidosis was observed in 95% of OHCA patients admitted to the ICU. Initial rhythm and time interval from collapse to ROSC were independently associated with base deficit. Base deficit was strongly associated with ICU mortality especially from refractory shock but seems to have no impact on neurological recovery in patients who were discharged alive. A substantial proportion of patients admitted with a very severe metabolic acidosis were discharged alive with a good neurological performance.

## Additional files

**Additional file 1: Fig. S1.** Base deficit distribution in the Study population. Red dashed lines represented 25%range interquartile, median and 75% range interquartile, respectively.

**Additional file 2: Table S1.** Characteristics of patients in the fourth base deficit quartile (base deficit > 13) according to vital status at ICU discharge.

**Additional file 3: Table S2.** Specificity and sensibility of base deficit, arterial blood lactate bicarbonate and pH level.

## Abbreviations

OHCA: out-of-hospital cardiac arrest
ICU: intensive care unit
ROSC: return of spontaneous circulation
BLS: basic life support
PCO_2_: partial pressure in carbon dioxide
PO_2_: partial pressure in oxygen
KDIGO: kidney disease: improving renal outcome
EMS: emergency medical service
ECG: electrocardiogram
CPC: cerebral performance category score
RRT: renal replacement therapy
