# Supplementary figures and images for: Severe metabolic acidosis after out-of-hospital cardiac arrest: risk factors and association with outcome
Source: Ann Intensive Care. 2018 May 8;8:62. doi: 10.1186/s13613-018-0409-3 (PMC5940999; doi:10.1186/s13613-018-0409-3)

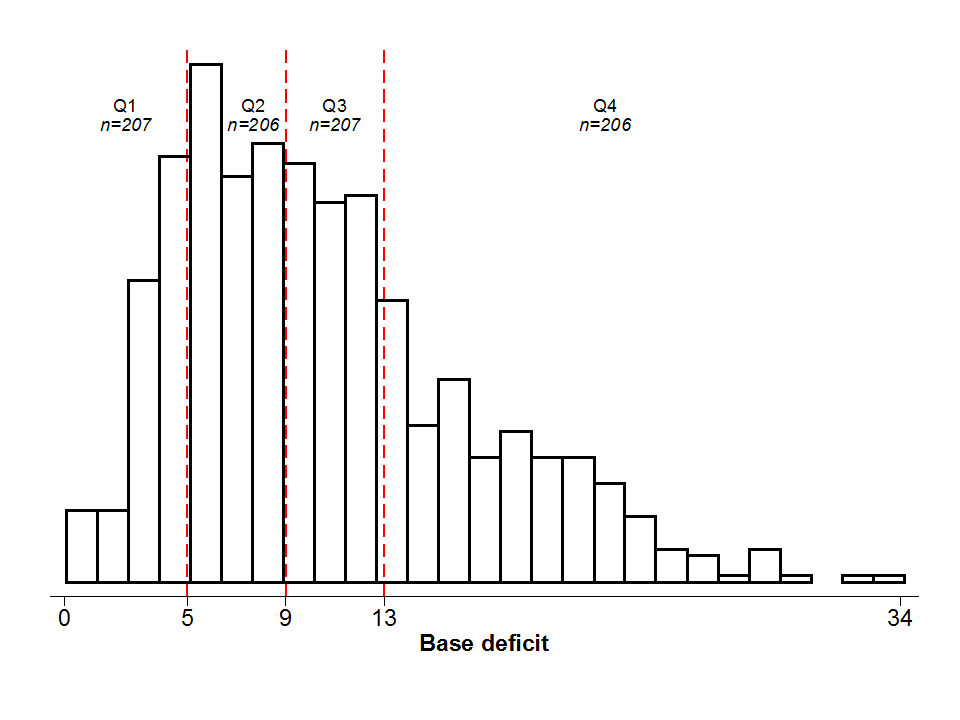

Supplement: Supplementary file 1 — Additional file 1: Fig. S1. Base deficit distribution in the Study population. Red dashed lines represented 25%range interquartile, median and 75% range interquartile, respectively. [file 13613_2018_409_MOESM1_ESM.tif]
