# Supplementary material for: Severe metabolic acidosis after out-of-hospital cardiac arrest: risk factors and association with outcome
Source: Ann Intensive Care. 2018 May 8;8:62. doi: 10.1186/s13613-018-0409-3 (PMC5940999; doi:10.1186/s13613-018-0409-3)
Supplement: Supplementary file 2 — Additional file 2: Table S1. Characteristics of patients in the fourth base deficit quartile (base deficit > 13) according to vital status at ICU discharge. [file 13613_2018_409_MOESM2_ESM.docx]

**Table S1. Characteristics of patients in the fourth base deficit quartile (base deficit > 13) according to vital status at ICU discharge**

| Variable | Discharged alive from ICU  n=24 | Dead within ICU stay  n=182 | p value |
| --- | --- | --- | --- |
| **Demographics** |  |  |  |
| Male gender | 14 (58.3) | 107 (58.8) | 0.966 |
| Age, y | 55.1 [38.7, 62.5] | 63.2 [53.1, 73.8] | 0.015 |
| **OHCA characteristics** |  |  |  |
| Public setting | 9 (37.5) | 33 (18.1) | 0.027 |
| Witnessed CA | 19 (79.2) | 138 (77.1) | 0.820 |
| Bystander CPR | 14 (63.6) | 87 (49.4) | 0.209 |
| Initial VF/VT | 18 (75.0) | 54 (29.7) | <0.001 |
| Collapse to ROSC, min | 30.0 [25.0, 37.0] | 28.0 [20.0, 40.0] | 0.961 |
| Prehosp. infusion of bicar. | 2 (8.3) | 44 (24.2) | 0.080 |
| Cardiac cause related CA | 14 (58.3) | 66 (36.3) | 0.08 |
| **Biological assessment at ICU admission** |  |  |  |
| pH | 7.09 [6.94, 7.14] | 7.02 [6.91, 7.11] | 0.169 |
| PCO_2_, mmHg | 42.9 [34.0, 51.8] | 43.5 [35.2, 53.0] | 0.929 |
| Bicarbonate level, mmol/l | 11.6 [9.9, 14.1] | 10.6 [8.5, 12.5] | 0.026 |
| Urea level, mmol/l | 6.5 [4.9, 7.9] | 8.6 [5.8, 14.0] | 0.007 |
| Creatinine level, µmol/l | 110 [102, 157] | 139 [101, 197] | 0.114 |
| Phosphorus level, mmol/l | 1.7 [1.2, 3.2] | 3.0 [2.2, 3.8] | 0.017 |
| Lactate level, mmol/l | 7.8 [5.0, 10.9] | 11.8 [8.4, 16.0] | 0.002 |
| Base deficit, mEq/L | 16.4 [14.3, 19.6] | 18.3 [15.3, 21.0] | 0.133 |
| **In-hospital characteristics** |  |  |  |
| Post resus. shock | 19 (79.2) | 146 (80.2) | 0.903 |
| Coronary angiography | 15 (62.5) | 111 (61.0) | 0.886 |
| Therapeutic hypothermia | 21 (87.5) | 143 (78.6) | 0.307 |
| RRT at day-1 | 16 (66.7) | 132 (72.5) | 0.548 |
| OHCA: out-of-hospital cardiac arrest; CPR: cardiopulmonary resuscitation; ROSC: restoration of spontaneous circulation; RRT: renal replacement therapy  Categorical variables are shown as n (%) and compared using a chi-square test or a Fischer test as appropriate  Continuous variables are shown as median [iqr] and compared using a Mann-Whitney's test | | | |
