# Supplementary material for: Severe metabolic acidosis after out-of-hospital cardiac arrest: risk factors and association with outcome
Source: Ann Intensive Care. 2018 May 8;8:62. doi: 10.1186/s13613-018-0409-3 (PMC5940999; doi:10.1186/s13613-018-0409-3)
Supplement: Supplementary file 3 — Additional file 3: Table S2. Specificity and sensibility of base deficit, arterial blood lactate bicarbonate and pH level. [file 13613_2018_409_MOESM3_ESM.docx]

**Table S2. Specificity and sensibility of base deficit, arterial blood lactate bicarbonate and pH level**

| **Parameter** | **Threshold** | **Specificity** | **Sensibility** |
| --- | --- | --- | --- |
| **Base deficit** | ≥ 25 mEq/L | 100% | 2.8% |
| **Arterial blood lactate level** | ≥ 20 mmol/L | 100% | 4.1% |
| **Arterial blood bicarbonate level** | ≤ 8.5 mmol/L | 100% | 8.7% |
| **Arterial pH** | ≤ 6.71 | 100% | 2.2% |
